# Phenylalanine as a hydroxyl radical-specific probe in pyrite slurries

**DOI:** 10.1186/1467-4866-13-3

**Published:** 2012-02-07

**Authors:** Shawn C Fisher, Martin AA Schoonen, Bruce J Brownawell

**Affiliations:** 1 School of Marine and Atmospheric Science, Stony Brook University, Stony Brook, NY 11794-5000, USA; 2 Department of Geosciences, Stony Brook University, Stony Brook, NY 11794-2100, USA

## Abstract

The abundant iron sulfide mineral pyrite has been shown to catalytically produce hydrogen peroxide (H_2_O_2_) and hydroxyl radical (**^.^**OH) in slurries of oxygenated water. Understanding the formation and fate of these reactive oxygen species is important to biological and ecological systems as exposure can lead to deleterious health effects, but also environmental engineering during the optimization of remediation approaches for possible treatment of contaminated waste streams. This study presents the use of the amino acid phenylalanine (Phe) to monitor the kinetics of pyrite-induced **^.^**OH formation through rates of hydroxylation forming three isomers of tyrosine (Tyr) - *ortho*-, *meta*-, and *para*-Tyr. Results indicate that about 50% of the Phe loss results in Tyr formation, and that these products further react with **^.^**OH at rates comparable to Phe. The overall loss of Phe appeared to be pseudo first-order in [Phe] as a function of time, but for the first time it is shown that initial rates were much less than first-order as a function of initial substrate concentration, [Phe]_o_. These results can be rationalized by considering that the effective concentration of **^.^**OH in solution is lower at a higher level of reactant and that an increasing fraction of **^.^**OH is consumed by Phe-degradation products as a function of time. A simplified first-order model was created to describe Phe loss in pyrite slurries which incorporates the [Phe]_o_, a first-order dependence on pyrite surface area, the assumption that all Phe degradation products compete equally for the limited supply of highly reactive **^.^**OH, and a flux that is related to the release of H_2_O_2 _from the pyrite surface (a result of the incomplete reduction of oxygen at the pyrite surface). An empirically derived rate constant, **K_pyr_**, was introduced to describe a variable **^.^**OH-reactivity for different batches of pyrite. Both the simplified first-order kinetic model, and a more detailed numerical simulation, yielded results that compare well to the observed kinetic data describing the effects of variations in concentrations of both initial Phe and pyrite. This work supports the use of Phe as a useful probe to assess the formation of **^.^**OH in the presence of pyrite, and its possible utility for similar applications with other minerals.

## Background

Reactive oxygen species (ROS) are highly reactive compounds that have been studied extensively in biological and environmental systems and have been linked to numerous human health issues, including Parkinson's disease [1] and lung cancer [2]. In addition to forming naturally in cells as a function of respiration [3] and in the atmosphere [4], recent studies have observed that ROS can form at the surface of some minerals in water [5-7]. In particular, pyrite (FeS_2_) has been shown to be efficient at forming hydrogen peroxide (H_2_O_2_) and hydroxyl radical (**^.^**OH) in the presence of oxygenated solutions [6,8]. Of significant interest in pyrite slurries is **^.^**OH, as it is transient and will rapidly react with any organic compound. The abundance of naturally-occurring pyrite in environments such as coal mines, where fine dust particles are frequently inhaled by workers, introduces the potential risk of human exposure to mineral-induced ROS. As an abundant mineral in many sediments and geological deposits, pyrite may also play a role in transformations of natural organic matter. Finally, the potential usefulness of pyrite as a tool for remediation of wastewater is also being considered as engineers continue to look for new methods of removing anthropogenic compounds before discharging effluent back into the environment.

Several mechanisms for surface-derived ROS in systems containing pyrite have been proposed that include iron-catalyzed, electron-transfer reactions. Schoonen et al. [6] suggests molecular oxygen is reduced to form H_2_O_2 _by reacting with iron-II (Fe(II)) sites at the pyrite surface. H_2_O_2 _may either remain adsorbed on the surface and be further reduced to **^.^**OH (Equation 1), or desorb and undergo Fenton chemistry with dissolved ferrous iron (Fe^2+^) to form **^.^**OH in solution (Equation 2); with pyrite dissolution acting as the source of Fe^2+^.

(1) $$\begin{array}{l} {\text{Fe(II)}}_{\left(s\right)}+{\text{H}}_{\text{2}}{\text{O}}_{\text{2}}{}_{\left(ad\right)}\to \\ \;{\text{Fe(III)}}_{\left(s\right)}+{\text{OH}}^{-}+\cdot {\text{OH}}_{\left(ad)\;\text{or}\;(aq\right)} \end{array}$$

(2) $$\begin{array}{l} {\text{Fe}}^{2+}{}_{\left(aq\right)}+{\text{H}}_{2}{\text{O}}_{2}{}_{\left(aq\right)}\to \\ \;{\text{Fe}}^{3+}{}_{\left(aq\right)}+{\text{OH}}^{-}+\cdot {\text{OH}}_{\left(aq\right)} \end{array}$$

The proportion of H_2_O_2 _that reacts to form **^.^**OH on the pyrite surface versus in solution is not known, although it has been proposed that the production of **^.^**OH and subsequent reactions with organic compounds occur primarily in the aqueous phase [6]. Alternatively, earlier work by Borda et al [8] hypothesizes that **^.^**OH can be formed directly from the oxidation of water by Fe(IV) defect sites on the pyrite surface. Although more recent studies suggest this pathway is much less important in the presence of dissolved oxygen, it remains a potential **^.^**OH source.

There have been a number of studies observing interactions with **^.^**OH and organic reactants in biological and ecological systems. Because direct measurement of **^.^**OH is not possible, a number of probes have been developed. In some studies **^.^**OH-specific phenyl-hydroxylation products of aromatic substrates have been employed [9]. In the case of pyrite slurries, probes that have been applied to determine **^.^**OH-flux include electron-spin resonance analysis with molecular traps [10] and fluorogenic probes such as 3'-(*p*-aminophenyl) fluorescein (APF) [11,12]. Cohn et al. [11] recently adapted the *in-vitro *APF method for use in pyrite slurries to quantify **^.^**OH. However, in order to measure **^.^**OH with APF, solution chemistry (e.g. pH) must be strictly controlled to prevent interference in fluorescence monitoring. Pyrite-mediated formation of **^.^**OH has also been implicated in RNA strand shortening [13] and oxidation of the nucleobase adenine to 8-oxoadenine [14]. Recent studies have shown that trichloroethylene (TCE) and its reaction products are degraded by pyrite (ultimately producing carbon dioxide (CO_2_)), implicating pyrite-derived **^.^**OH as the main oxidant [15,16]. Contributions such as this have led to an interest in the potential use of pyrite in engineered systems to facilitate remediation of organic chemicals in waste stream. However, the TCE degradation products identified are not necessarily specific to ROS reactions, and alternative pathways (e.g. microbial) may yield similar products in the environment [17,18]. Thus there is a need for a probe to study reactions in unconstrained mineral systems that can be monitored over time with **^.^**OH-specific products, and is relevant to address a range of human health and environmental concerns.

**Figure 1:**
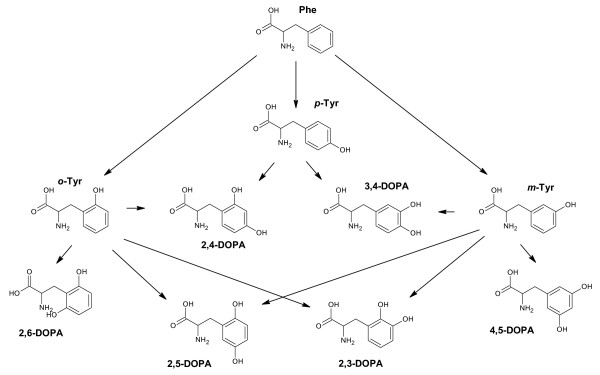
**Potential oxidation pathways of Phe and Tyr isomers**. In this study, phenyl hydroxylation of Phe by **^.^**OH has been shown to form *o*-, *m*-, and *p*-Tyr. Subsequent degradation of Tyr isomers are the six different isomers of DOPA.

The fate of phenylalanine (Phe) and its degradation products were investigated in this work as a potential probe to examine pyrite-mediated **^.^**OH reactions. As a naturally occurring amino acid, Phe has been shown to undergo **^.^**OH-specific phenyl hydroxylation reactions to form *ortho-*, *meta-*, and *para-*tyrosine (*o*-Tyr, *m*-Tyr, and *p*-Tyr) [19] as depicted in Figure 1. The *o*-and *m*-Tyr isomers have been used to monitor oxidative stress both *in-vitro *and *in-vivo *[20] as they do not form during normal biological processes. The Tyr isomers are stable enough to be measured in urine and proteins [21-24]. Analysis of Phe and the Tyr isomers can be observed simultaneously and at low levels (tens of nanomolar) with HPLC-MS methods, providing usefulness as a biologically-relevant probe. Additionally, isomers of Tyr can oxidize in the presence of **^.^**OH to various isomers of dihydroxylphenylalanine (DOPA) (Figure 1), which may provide additional insight into the fate of Phe in mineral slurries as only 3,4-DOPA is naturally formed as the primary product through biological transformation of *p*-Tyr [25,26].

This work describes the development of a sensitive HPLC-MS based method to evaluate the use of Phe and its reaction products as a way to probe **^.^**OH-specific reactions involving pyrite in aqueous solution. The method was applied to study the kinetics of observed reactions as a function of both pyrite loading and concentration of Phe in order to gain additional insights into processes that control reaction rates. As had been described in earlier studies with other organic compounds [6,16], the loss of Phe in an individual experiment could be adequately represented as pseudo first-order as a function of time. However, the effect of initial concentration of reactant had not been examined, and an unanticipated dependence on initial concentration of Phe ([Phe]_o_) was observed. A conceptual model is presented using a number of simplifying assumptions that incorporates the combined effects of pyrite loading and [Phe]_o _on **^.^**OH levels in bulk solution (e.g. competition reactions between Phe and its degradation products), allowing for interpretative comparisons between predicted and observed data for both Phe and Tyr.

## Experimental Methods

### Materials

Pyrite from Huanzala, Peru (Wards Natural Science, Rochester, NY) was ground and sieved to a range of 38 - 63 μm with a surface area of 1.25 m^2^/g as per Cohn, et al. [14]. Phenylalanine, *para*-tyrosine, *ortho*-tyrosine, 3,4-dihydroxyphenylalanine, and beta-mercaptoethanol (β-ME), all 99% ACS-grade or better, were obtained from Alfa Aesar (Ward Hill, MA). *meta*-Tyrosine was obtained from TCI America (Portland, OR). Methanol was GC^2^-grade from Burdick & Jackson (Morristown, NJ). Formic acid was ACS-grade from EMD Chemicals (Gibbstown, NJ). All water used for cleaning, standards, reactions, dilutions, and chromatography work was purified with a Milli-Q filtration system (Millipore Corporation, Billerica, MA) to a resistivity of 18.3 MΩ/cm.

### Phenylalanine degradation Experiments

Fresh samples of crushed pyrite were treated prior to each experiment with a nitrogen-purged solution of hydrochloric acid to remove surface oxidation and rinsed in a glove box with nitrogen-purged water, as per Cohn et al. [14]. The pyrite was kept in a sealed vial and removed from the glove box no-more than several hours prior to the experiment. Aqueous stock solutions of Phe (or Tyr) were prepared under ambient room conditions, diluted to desired initial concentrations, and added to pyrite in 15 or 50 mL disposable centrifuge tubes to initiate the reaction (leaving 25 - 30% of the volume as oxic headspace). Total volume of each reaction mixture was always at least 4-times that of the combined volume of aliquots sequentially removed during kinetic studies. Tubes were then immediately set to rotate end-over-end on a carousel at a constant 24 rotations-per-minute (the minimum rate found to fully suspend the slurry) and covered with aluminum foil to prevent light exposure. All experiments were conducted at room temperature of 25° ± 3° Celsius.

At predetermined time points, tubes were briefly removed from the carrousel, briefly vortexed, and a 300 μL aliquot was removed with a 1 mL Eppendorf^® ^pipette at the same pyrite-to-water ratio as the sample (determined by a mass-balance test of repetitive sampling). Samples, including controls, were then quenched with 5 μL of β-ME (for a concentration of 234 mM) and filtered with 0.22 μm nylon Costar^® ^centrifuge vial filters (Corning Life Sciences, Lowell, MA). Portions of the filtrate were then diluted (to different extents based on their initial concentrations) with water directly in a 2 mL HPLC vial. Methanol (5%) and formic acid (0.5%) were also added to each vial to match initial mobile phase conditions.

Each experiment was conducted independently and consisted of sets of incubations that ran concurrently for a predetermined length of time. Seven individual experiment sets (A - G) were conducted for this study. Table 1 lists the conditions for each experiment including incubation time, [Phe]_o _levels, and pyrite loadings. For the majority of the experiments conducted, pyrite was added at 100 g/L levels. The pH was monitored during three sets of experiments (Table 1) and was observed to drop rapidly within the first several minutes of incubation, after which it remained in a narrow range over the time-course of the experiment (pH 2.6 - 2.9) when pyrite loading was ≥50 g/L. When less pyrite was added, the final pH of these unbuffered solutions was higher (pH 4.2 - 5.5; Table 1), increasing as pyrite loadings decreased from 25 to 5 g/L.

**Table 1:** Experimental Design

Experiment Set	[Phe]_o_	[pyr]	Incubation time	pH
	(μM)	(g/L)	(hr)	
A	26.6	100		2.5
	102.8	100	72	2.6
	306.7	100		2.5
**B**	**12.5**	**100**		
	**37.1**	**100**		
	**97.8**	**100**	**120**	
	**401.5**	**100**		
	**1127**	**100**		
C	1	100		
	3	100	6-hour pyrite incubations + 1 hr pyrite and Phe	
	10	100		
	30	100		
	100	100		
**D**	**10.9**	**25**		
	**11.4**	**50**		
	**11.4**	**100**		
	**28.5**	**25**		
	**29.5**	**50**		
	**28.6**	**100**	**12**	
	**93.9**	**25**		
	**91.9**	**50**		
	**94.5**	**100**		
	**285.2**	**25**		
	**281.4**	**50**		
	**285.6**	**100**		
E	8.7	5		5.5
	9.4	10		5.1
	9.3	25	16	4.2
	10.4	50		2.9
	10.4	100		2.6
**F**	**81.68**	**10**	**72**	
	**107.2**	**10** **(14 m^2^/g)**	**240**	
G	98.8	50		2.8
	[*o*-Tyr]_o _94.0	50	72	
	[*m*-Tyr]_o _97.6	50		
	[*p*-Tyr]_o _97.7	50		

Experiments A - G listed above with corresponding [Phe]_o_, initial pyrite loading, incubation time of the experiment, and pH data. The pH was not monitored in every reaction vessel for each experiment as it was not a variable as all (Exp. B, C, F, and G) or multiple vials (Exp. D) had the same pyrite loadings. Reaction vessels in experiment G contained only one reactant each - Phe, *o*-Tyr, *m*-Tyr, or *p*-Tyr. Pyrite was added to the respective dilutions of Phe (or Tyr) to initiate the reaction.

### Instrumental Analysis

Instrumental analyses were conducted on a Waters Corporation (Milford, MA) Alliance^® ^2695 HPLC coupled to a Waters Corporation Micromass LCT Time-of-Flight Mass Spectrometer (ToF-MS). A Phenomenex (Torrance, CA) Luna^® ^C18(2) HPLC column 3 mm × 250 mm with 5 μm particle size was heated to 40°C and run with a gradient containing methanol (Solvent A) and 10 μM ammonium formate/formic acid in water (pH 3.5) (Solvent B). Total run time was 16 minutes with a gradient of: 10% to 70% Solvent A over 8 minutes; 70% to 10% in 4 minutes; followed by a re-equilibration time of 4 minutes. A solution of Leucine enkephalin (Sigma Aldrich, St. Louis, MO) was injected post-column, generally at 1 - 3 μL/min, for internal mass calibration. Mass spectrometer parameters were 2800 V for the capillary voltage in positive-ion, electrospray (ESI+) mode with cone and extraction voltages set at 20 V and 3 V respectively. Calibration standards containing Phe, *o*-, *m*-, *p*-Tyr, and sometimes DOPA (with concentrations ranging from 100 nM to 5 μM) were run with each sample set with method detection limits for Phe and Tyr of 50 nM (as determined by a signal-to-noise ratio of 3:1). Accurate mass measurements of analytes in both standards and samples were calculated to be within 2 mDa of the actual M+H^+ ^mass with spectral resolutions between 5000 - 6000 for all experiments.

### Controls

**Figure 2:**
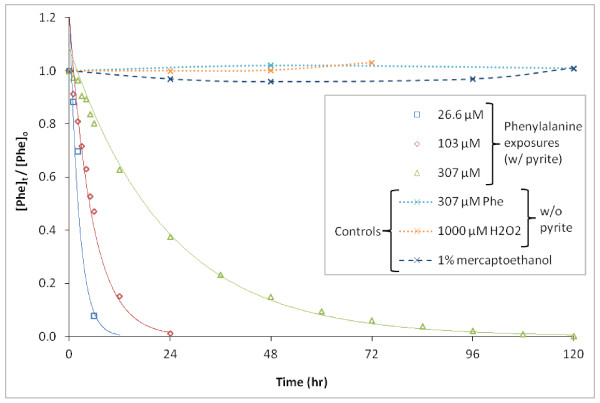
**Degradation of various Phe concentrations in the presence of 100 g/L pyrite (Exp. A)**. Phe concentrations through time are fit well with exponential regressions. Controls of 307 μM Phe are also shown without pyrite in pure water and 112 μM Phe in 1000 μM H_2_O_2_; as well as with pyrite quenched with 0.1% (14.2 mM) β-ME.

Excess β-ME was added for controls in several experiments as a quenching reagent; the reported second-order rate constant for β-ME and **^.^**OH is 6.9 × 10^9 ^M^-1 ^s^-1 ^[27], similar to 6.5 × 10^9 ^M^-1 ^s^-1 ^for Phe and **^.^**OH [28]. No loss of Phe from solution was observed when 0.1% (14.2 mM) β-ME was added to pyrite slurries (Figure 2). This provides evidence that the observed loss of solution-phase Phe was not due to adsorption of Phe to pyrite. Additional control incubations of Phe in water without pyrite accompanied each experiment and always resulted in complete recoveries, indicating that there were no other losses of Phe (including enzymatic reactions in non-sterile media) occurring in this study.

Production of H_2_O_2 _has been measured in prior studies with similarly prepared pyrite slurries [29]. No loss of Phe over 72 hours was observed in pyrite-free solutions of H_2_O_2 _added at a wide range of concentrations (10 μM, 100 μM, and 1000 μM) (Figure 2); nor was Tyr production observed. The H_2_O_2 _level shown in Figure 2 is far greater than the range found to be produced in 160 g/L pyrite slurries, which varied from undetectable in the absence of the iron chelator ethylenediaminetetraacetate (EDTA) and up to 26 μM in the presence of EDTA [29]. These observations indicate that there was no direct reaction between Phe and H_2_O_2_, no measurable losses to adsorption of Phe to pyrite, and no microbial degradation (which would have resulted in a coincidental loss of Phe and preferential formation of *p*-Tyr).

## Results

### Kinetics of Phenylalanine loss

The kinetics of Phe degradation in aqueous suspensions of pyrite were highly dependent on the [Phe]_o _and the amount of pyrite mineral surface in solution. The observed effects of the [Phe]_o _proved to be interesting and were examined in four experiments conducted under different conditions (Exp. A - D; Table 1). The results from these studies are illustrated in Figures 2 through 4. In each experiment the timescale for the disappearance of Phe was seen to increase with increasing [Phe]_o_. As shown in studies conducted with pyrite and TCE [16] or adenine [6], loss of Phe as a function of time is described well as pseudo first-order (Equation 3); however, prior studies did not examine the effect of initial concentrations of Phe and pyrite loadings.

(3) $$-\frac{\text{d}[\text{Phe}]}{\text{dt}}=k\prime [\text{Phe}]$$

**Figure 3:**
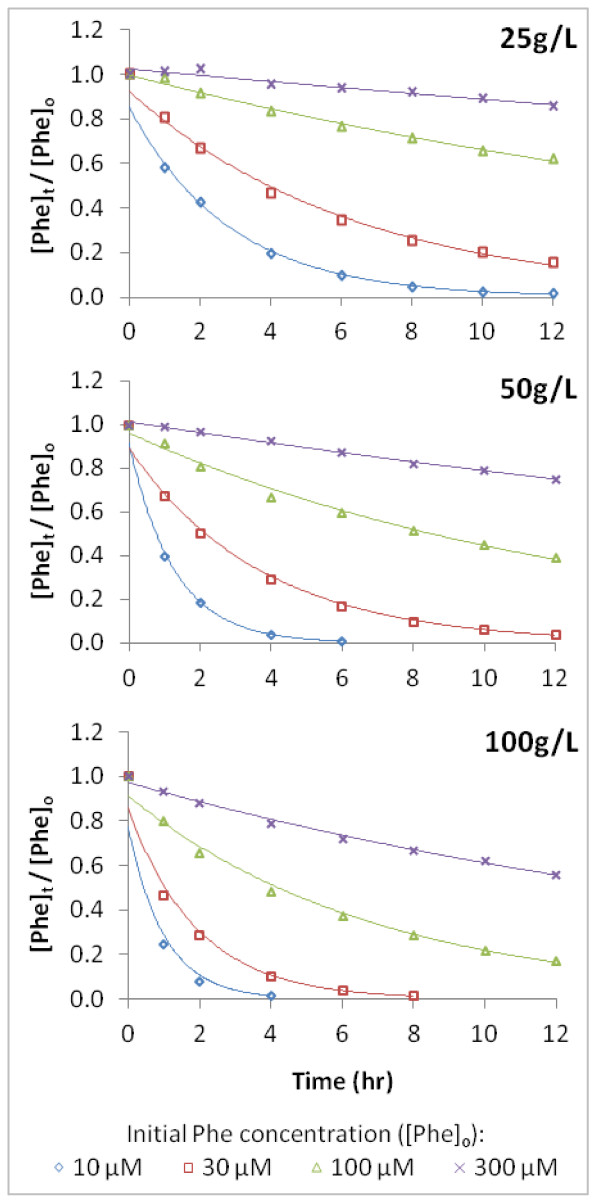
**Concentration of Phe over time as a function of pyrite loadings (Exp. D)**. Data is normalized to [Phe]_o_. For each pyrite loading, incubations of [Phe]_o _from 10 μM to 300 μM were monitored for 12 hours. Data points for Phe are compared with fits to exponential regressions corresponding to a first-order model.

Where ***k' ***is the pseudo first-order rate constant derived through a fit of the data between the start of the reaction and when 90% of the Phe was lost (when reaction times permitted) (see fits in Figures 2 and 3). Calculated ***k' ***values were inversely related to [Phe]_o _as illustrated in Figure 2 (Exp. A) with values of 0.45 hr^-1^, 0.13 hr^-1^, and 0.04 hr^-1 ^for initial concentrations 26.6 μM, 103 μM, and 307 μM, respectively. Observed data was well described by Equation 3 (Figure 1) with correlation (R^2 ^= 0.972 ± 0.031) provided in Table 2.

**Table 2:** Kinetic values for experimental sets of data for this study

Experiment Set	[Phe]_o_	[pyr]	*R_o_*	*k'*	Obs. half-life	Calc. half-life	half-life	ln[Phe]/t
	(μM)	(g/L)	(μM/hr)	(hr^-1^)	(hr)	(hr)	Ratio	**R^2 ^- correl**.
A	26.6	100	4.01	0.447	3.3	1.6	0.47	0.9627
**K_pyr _**= 0.122 μmol g^-1 ^hr^-1^	103	100	9.32	0.157	5.5	4.4	0.80	0.9849
	307	100	10.3	0.040	18.0	17.3	0.96	0.9994
**B**	**12.5**	**100**	**1.43**	**0.642**	**3.5**	**1.1**	**0.31**	**0.9293**
**K_pyr _**= 0.079 μmol g^-1 ^hr^-1^	**37.1**	**100**	**3.17**	**0.199**	**6.0**	**3.5**	**0.58**	**0.9498**
	**97.8**	**100**	**3.63**	**0.093**	**14.0**	**7.5**	**0.53**	**0.9717**
	**402**	**100**	**4.69**	**0.025**	**35.0**	**27.7**	**0.79**	**0.9768**
	**1130**	**100**	**4.57**	**0.008**	**90.0**	**86.6**	**0.96**	**0.9422**
C	1	100	0.79	--	--	--	--	--
	3	100	2.05	--	--	--	--	--
	10	100	5.65	--	--	--	--	--
	30	100	10.2	--	--	--	--	--
	100	100	16.3	--	--	--	--	--
**D**	**10.9**	**25**	**^+^4.59**	**0.400**	**1.5**	**1.7**	**1.16**	**0.9917**
**K_pyr _**= 0.165 μmol g^-1 ^hr^-1^	**11.4**	**50**	**^+^6.86**	**0.844**	**0.8**	**0.8**	**1.03**	**0.9969**
	**11.4**	**100**	**^+ ^8.57**	**1.39**	**0.7**	**0.5**	**0.71**	**--**
	**28.5**	**25**	**4.74**	**0.156**	**3.6**	**4.4**	**1.23**	**0.9935**
	**29.5**	**50**	**9.53**	**0.283**	**2.0**	**2.4**	**1.22**	**0.9975**
	**28.6**	**100**	**^+^15.3**	**0.559**	**0.9**	**1.2**	**1.38**	**0.9915**
	**93.9**	**25**	**4.15**	**0.041**	**16.8**	**16.9**	**1.01**	**0.9907**
	**91.9**	**50**	**8.65**	**0.077**	**8.5**	**9.0**	**1.06**	**0.9909**
	**94.5**	**100**	**18.9**	**0.143**	**3.8**	**4.8**	**1.28**	**0.9932**
	**286**	**25**	**3.83**	**0.014**	**38.6**	**49.5**	**1.28**	**0.9426**
	**282**	**50**	**6.45**	**0.025**	**23.2**	**27.7**	**1.19**	**0.9942**
	**286**	**100**	**17.1**	**0.047**	**15.1**	**14.7**	**0.98**	**0.9916**
E	8.7	5	0.11	0.017	28.0	40.8	1.46	0.8834
**K_pyr _**= 0.021 μmol g^-1 ^hr^-1^	9.4	10	0.2	0.042	16.5	16.5	1.00	0.9611
	9.3	25	0.40	0.092	10.0	7.5	0.75	0.9000
	10.4	50	0.84	0.130	6.6	5.3	0.81	0.9529
	10.4	100	1.38	0.174	4.5	4.0	0.89	0.9880
**F**	**81.7**	**10**	**0.88**	**0.007**	**70**	**99.0**	**1.41**	**0.9863**
	**107**	**10** **(14 m^2^/g)**	**9.38**	**0.116**	**6**	**6.0**	**1.00**	**0.9993**
G	98.8	50	15.1	0.079	5.5	8.8	1.59	0.9854
	[*o*-Tyr]_o _94.0	50	14.9	0.081	5.5	8.6	1.56	0.9941
	[*m*-Tyr]_o_97.6	50	21.1	0.166	3	4.2	1.39	0.9945
	[*p*-Tyr]_o _97.7	50	21.2	0.163	3	4.3	1.42	0.9922

					**Average R^2 ^(sets A, B, D, E, F)**			**0.97 ± 0.03**

Data for ***R*_o _**determined where approximately 20% of initial Phe was degraded. ***k' ***was estimated from time points up to 90% of Phe loss when possible, and varies between experiment sets due to variability in pyrite activity relative to **^.^**OH production. Set **C **was a single-time point experiment and thus the data do not yield relevant ***k' ***values or half-lives. ^+ ^= ***R*_o _**was estimated as Phe loss was greater than 30% by the first time point.

Figure 2 also illustrates other important observations from this study. Experiment A was one of several experiments where a high mass loading of pyrite was added (100 g/L). At the highest [Phe]_o_, 307 μM, degradation continues for as long as 120 hours, consistent with evidence for long-term production of H_2_O_2 _and **^.^**OH obtained in prior studies with pyrite conducted for up to several weeks under similar conditions [14]. Also, initial rates, ***R*_o _**(μM/hr), varied non-linearly as a function of [Phe]_o_. Values of ***R*_o _**were estimated to be 4.1 μM/hr, 9.6 μM/hr, and 10.3 μM/hr for [Phe]_o _of 26.6 μM, 103 μM, and 307 μM respectively (Figure 2). ***R*_o _**was estimated by linear regression of data collected at **t **= 0 through the time at which [Phe] is approximately 80% of initial values.

A similar dependence of ***R*_o _**on [Phe]_o _was also observed in experiments A - D, even at different pyrite loadings (Exp. D) (Table 2). In experiment D, incubations on a shorter timescale (12 hours) were monitored at a greater frequency for better resolution of Phe loss (Figure 3). The loss of Phe was determined under conditions where both [Phe]_o _(11 - 286 μM) and pyrite loading (25 - 100 g/L) were varied (the effect of pyrite loading will be addressed later in this section).

**Figure 4:**
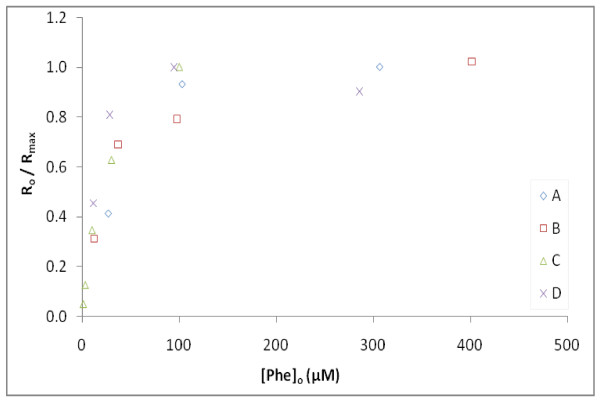
**Initial degradation rates of Phe as a function of [Phe]_o_**. Shown is ***R*_o _**normalized to maximum initial degradation rates (***R*_max_**) for each experiment set (hypothesized to vary due to subtle differences in pretreatment of pyrite). Pyrite loadings of 100 g/L were used in all experiments. ***R*_max _**for each experiment set: **A **is 10.3 μM/hr, **B **is 4.69 μM/hr, **C **is 16.3 μM/hr, **D **is 18.9 μM/hr. Results illustrate the lack of first-order dependence of degradation as a function on the disappearance of Phe.

Figure 4 shows the same relationship between the initial rates for each incubation from experiments A - D as a function of [Phe]_o _when normalized to a maximum rate determined for each respective experiment (***R*_o _**/***R*_max_**) are combined (all conducted at 100 g/L pyrite loading). This normalization of ***R*_o _**accounted for differences between ***R*_max _**values determined in experiments conducted on different days (Table 2). While pyrite samples were all derived from the same homogenized batch of ground and sieved mineral, the explanation for variability in the reactivity of pyrite was not determined, but hypothesized to have resulted from subtle differences in acid pretreatment conditions that affected the abundance of Fe(III) surface sites. As further seen below, when results of different experiments with different samples of pyrite were combined, measured observations could be described by the same mechanistically-based kinetic model.

Rates increased proportionally with increasing concentration below 30 μM and clearly plateau at higher [Phe]_o_. Estimates of ***R*_o _**at lower [Phe]_o _were based on few data points where Phe had already degraded appreciably, and thus estimates of ***R*_o _**are more uncertain and underestimate the true initial rate to a greater extent. Still, the results in Figure 4 follow a hyperbolic relationship, which has often been interpreted using the Langmuir-Hinshelwood (L-H) equation when describing rates of reactant loss with catalysts (e.g. UV-irradiated titanium dioxide (TiO_2_)) that generate **^.^**OH at the metal surface [30,31]. Alternatively, it is proposed here that observed kinetics of the Phe data are more likely due to changes in [**^.^**OH]_(*aq*) _controlled simultaneously by the rate of H_2_O_2 _production at the pyrite surface and **^.^**OH interaction with Phe and its products (a detailed explanation can be found in the Discussion section).

**Figure 5:**
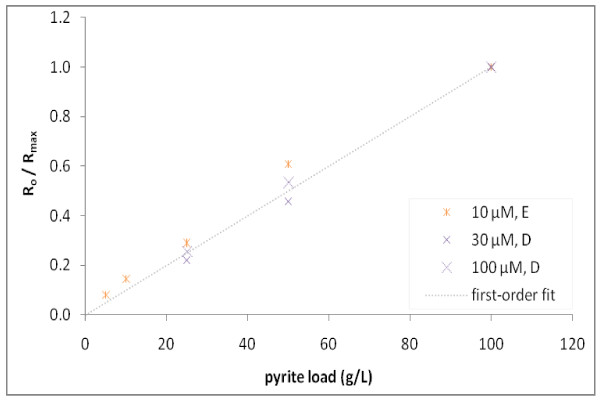
**The effect of pyrite loading on relative rates**. Shown is ***R*_o _**normalized to maximum initial degradation rates (***R*_max_**) combining data where pyrite loading was varied for three [Phe]_o_: 10 μM (Exp. E); and 30 μM and 100 μM (Exp. D). ***R*_max_**) was greatest at highest pyrite loading but varied among the three experiments due to the effect of [Phe]_o _on ***R*_o _**(Figure 4) and differences in pyrite reactivity between experiments D and E (see Table 2).

**Figure 6:**
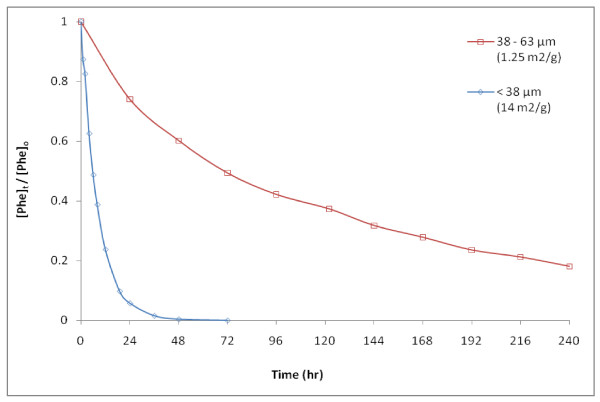
**Effect of surface area on the rate of Phe degradation**. Loss of 100 μM [Phe]_o _in 10 g/L pyrite with surface areas were 1.25 m^2^/g for 38 - 63 μm (size used in all other experiments) and 14 m^2^/g for < 38 μm particle sizes.

The effects of varying pyrite loading and surface area (which affect **^.^**OH production) on Phe degradation rates are evident in Figures 3, 5, and 6. Increased pyrite loadings of 25 g/L up to 100 g/L resulted in proportional increases in ***R*_o _**and ***k' ***at the same [Phe]_o _(Figure 3; Table 2). Figure 5 illustrates that rates (as ***R*_o _**/***R*_max_**) versus pyrite loading for several different [Phe]_o _have first-order dependence on pyrite loading. Although the effect of pH on Phe degradation was not studied for this work, it is noteworthy to point out that ***R*_o _**determined at the lowest pyrite loading (pH = 5.5 at 5 g/L, Table 1 (Exp. E)) was easily measured and still linearly correlated with those determined at higher pyrite loadings (pH = 2.9 and 2.6 at 50 and 100 g/L pyrite, Table 1 (Exp. E)). Interestingly, the initial rates of H_2_O_2 _formation in similar systems have been observed to be relatively unaffected over a range of pHs [6]. Because many steps leading to the formation and fate of H_2_O_2_, **^.^**OH, and ferrous iron have the potential to be pH-dependent, more tests are needed to better understand the catalytic properties of pyrite as a function of pH.

Figure 6 illustrates the relationship between Phe loss and the surface area of pyrite in solution (Exp. F). At the same pyrite loading of 10 g/L, ***R*_o _**increased from 0.88 μM/hr to 9.4 μM/hr when the surface area was increased from 1.25 m^2^/g (38 - 63 μm) to 14 m^2^/g (< 38 μm fraction) respectively (Table 2), using pyrite that passed through the 38-μm sieve following initial particle-size separation. The difference in Phe loss between the two incubations equates to approximately a 10-fold increase in estimated ***R*_o _**for a corresponding 11-fold increase in pyrite surface area and thus is also consistent with first-order kinetics in pyrite surface area. Additionally, Figure 6 shows the Phe loss continuing for 10 days, twice the timescale in experiment A.

### Tyrosine production and loss resulting from phenylalanine degradation

**Figure 7:**
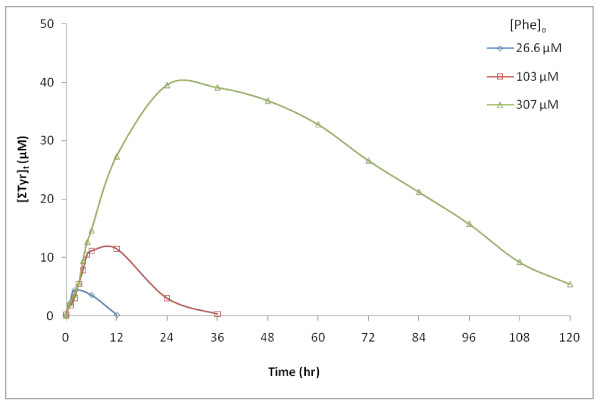
**Concentration of ΣTyr relative to [Phe]_o_**. The time-course of Tyr in experiment A, corresponding to the loss of Phe, illustrated in Figure 2. Accumulation reaches a maximum of 15% ± 3% of the initial Phe concentration at time points immediately following the half-life of Phe, after which degradation of Tyr exceeds its production.

The hydroxylation products of Phe were readily measured as *o*-, *m*-, and *p*-Tyr in these pyrite incubation studies (Figure 7). The formation of the three isomers of Tyr has been attributed to ^.^OH-specific reactions, and was not observed to occur with H_2_O_2_. The production of the sum of the three Tyr isomers (ΣTyr) initially occurs at rates that correlate with changes in initial rates of loss of Phe. Figure 7 illustrates the [ΣTyr] through time in experiment A, corresponding with losses of Phe shown in Figure 2. Similar to ***R*_o _**for Phe results, the initial increase in Tyr levels (from 2.0 to 2.5 μM/hr) is relatively independent of [Phe]_o_, contrary to expectations of reactions that are first-order in reactants. As the reactions proceed, ΣTyr levels peak then decrease over time. This would be expected as the production rate of Tyr drops due to less production from decreasing [Phe], combined with expected concurrent reactions of Phe and Tyr competing for the same available **^.^**OH pool.

**Figure 8:**
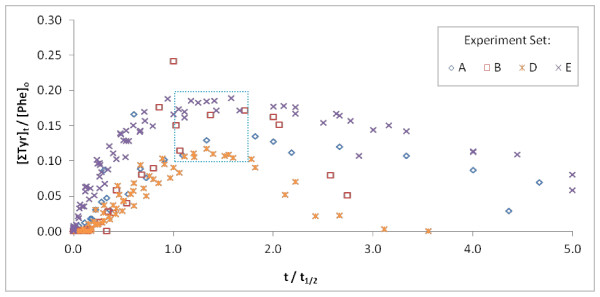
**Time-dependence of ΣTyr relative to [Phe]_o _across all conditions of variable Phe and pyrite levels**. The [ΣTyr] through time relative to the [Phe]_o _peaks within a narrow range (shown in the boxed area) corresponding to similar timescales relative to Phe loss. The differences that are apparent in the kinetics of Tyr decay between experiments are due to variations in pyrite reactivity, but predicted by model calculations show below.

The highest levels of [ΣTyr] always peaked at time points occurring near to or just beyond the half-lives of Phe, and within a relatively narrow percentage of [ΣTyr]/[Phe]_o_, typically between 12% and 18% (Figure 8). There is especially good agreement when [ΣTyr] versus Phe half-life is plotted for incubations with the same sample of pyrite. Results agree with predictions from the quantitative model developed and presented below.

Ratios of the three Tyr isomers were observed to be the same among all experiments, and thus consistent with a common mechanism for conversion of Phe to Tyr independent of [Phe]_o _or pyrite loadings. Random hydroxylation of the five aromatic reaction sites on Phe would result in a ratio of 2:2:1 for *o*-, *m*-, and *p-*Tyr isomers, respectively. Illustrative of other experiments from this study, data normalized to [ΣTyr] shows that *o*-, *m*-, and *p-*Tyr were relatively constant over time in experiments with variable pyrite loading with ratios of 0.40 (± 0.02) : 0.28 (± 0.02) : 0.32 (± 0.02) respectively (see Additional File 1, Figure S1). These results show that Tyr products are not formed at equal-molar concentrations as hypothesized elsewhere [19] or at a 2:2:1 ratio. Instead, the electrophilic attack of the **^.^**OH appears directed to the *para *position over the *ortho *and *meta *positions. In experiments where **^.^**OH was produced via pulse-radiolysis, *ortho*- and *para*- directed hydroxylation of toluene resulted in *o*-, *m*-, *p*-cresol ratios of 0.48 : 0.23 : 0.29, respectively [32]. More importantly, the Tyr-isomer ratios in pyrite slurries agree with those observed in homogeneous solutions containing variable levels of Fenton reagents (H_2_O_2 _and Fe^2+^) (data not shown). This finding is consistent with mechanisms where hydroxylation of Phe occurs in the aqueous phase, rather than a surface reaction where molecular orientations of adsorbed species may lead to changes in reaction product yields.

**Figure 9:**
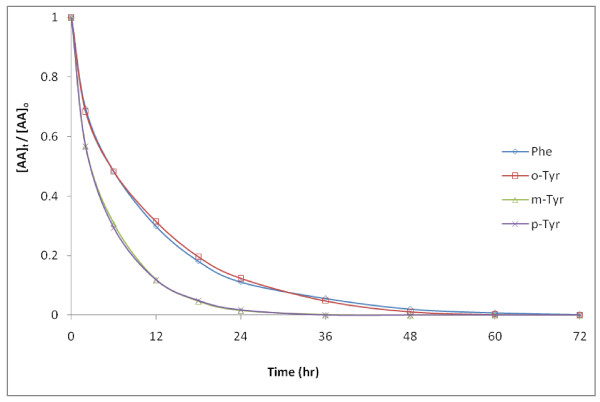
**Degradation of 100 μM initial concentrations of Phe, *o*-, *m*-, and *p*-Tyr in 50 g/L pyrite (Exp. G)**. The initial rate of *m*- and *p*-Tyr loss is 1.4 ± 0.1 times faster than that of Phe and *o*-Tyr, likely due to differences in the distribution of electron-density in the phenyl group.

The degradation of individual Tyr isomers was compared to that of Phe in experiment G (Figure 9; Table 2). Reaction rates for *m*- and *p*-Tyr were very similar to each other with an estimated ***k' ***of 0.17 hr^-1^, which were faster than those determined for *o-*Tyr and Phe (approximately 0.08 hr^-1^). Estimates for ***R*_o _**for each reactant were closer; 22 μM/hr for *m*- and *p- *Tyr and 15 μM/hr for *o-*Tyr and Phe. These modest differences in rates between Phe and Tyr are consistent with published second-order rate constants with **^.^**OH of 1.3 × 10^10 ^M^-1 ^s^-1 ^for *p-*Tyr and 6.5 × 10^9 ^M^-1 ^s^-1 ^for Phe (solution at pH = 2 for each) determined by pulse radiolysis [28]. Thus Tyr can be expected to compete effectively with Phe with an average rate (for the 3 isomers) that is approximately 1.3 to 1.7 times faster.

The production of DOPA as a phenyl-hydroxylation product of Tyr was also observed in this study. DOPA was identified by correspondence of HPLC retention with an authentic standard of one of the possible isomers (3,4-DOPA) which eluted before *p*-Tyr, and further confirmed by accurate mass measurements by Tof-MS that were within 2 mDa of the actual mass. This finding is consistent with production of DOPA for reactions of Tyr and **^.^**OH in prior work [26]. No other peaks corresponding to the mass of DOPA were identified. Due to the very low concentrations of DOPA (when detected) in Phe experiments at the dilution-levels injected, it was not routinely monitored. It is noteworthy that in an experiment with *p*-Tyr, DOPA was only measured at a maximum concentration around 2% relative to [*p*-Tyr]_o_. Although a comprehensive HPLC-MS method to chromatographically separate, indentify, and quantify the 6 possible isomers of DOPA was not conducted, it appears that Tyr-to-DOPA may not be as sensitive of a probe as Phe-to-Tyr in monitoring **^.^**OH-specific reactions mediated by pyrite.

## Discussion

### Phenylalanine as a hydroxyl radical probe

A sensitive method was developed for the quantification of Phe and Tyr at ≥50 nM levels by HPLC-MS methods with direct aqueous injection without the need for pre-concentration or derivatization steps that could lead to more analytical uncertainty. The role of **^.^**OH in the degradation of Phe is confirmed by the production of a characteristic composition of three isomers of Tyr - *ortho*, *meta*, or *para*. The presence of *m*- and *o*-Tyr has been used previously to assess the importance of **^.^**OH in oxidative stress [20]; whereas observation of primarily *p*-Tyr is normal in biological systems. High yields, characteristic ratios, and persistence of readily-measured Tyr products are other traits that make Phe a good probe to monitor **^.^**OH production in pyrite and other mineral slurries. Yields of Phe to ΣTyr conversion were estimated in this study to be between 30% and 60% by extrapolating initial changes in the [ΣTyr]_t_/([Phe]_o _- [Phe]_t_) back to time-zero (data not shown). The stability in pyrite slurries of Tyr is similar to that of Phe, allowing it to be monitored at appreciable levels throughout the period of Phe decay.

There are of course other molecular probes that have been developed that rely upon measurement of phenyl hydroxylation products to determine concentration levels of **^.^**OH in aqueous solutions. Two of the more sensitive ones are production of hydroxybenzoic acids from benzoic acid [9] and hydroxyterephthalic acid from terephthalic acid [33]. In each case, the determination of products by fluorescence or HPLC-fluorescence can be even more sensitive and require less expensive instrumentation than the HPLC-MS based detection of Phe and Tyr described here. However, neither benzoic acid nor terephthalic acid are nearly as fluorescent as their products. Hence, in experiments with those probes only the hydroxylated products are typically measured, while the substrate - often added in excess - is routinely not analyzed for. When detection of these substrates is required, it is usually conducted with far less sensitive UV-based methods [34].

There are other potential advantages of developing Phe as a probe to study **^.^**OH-mediated reactions. The presence of Phe in cells and body fluids allows for meaningful interpretation of the ratios of *o*-, *m*-, *p*-Tyr *in vivo *[21,23]. Similarly, in contrast to other aromatic probes used to date, Phe is naturally present at low levels in all natural waters and biologically active geological matrices, including environments where pyrite is present. With methods available for determining ultra-trace environmentally relevant concentrations of Phe and Tyr isomers [35,36], it may be possible to conduct field studies that could shed insight into the conditions where **^.^**OH-mediated reactions are occurring in the environment.

The mass spectrometric methods utilized also provide certain advantages. With the full-spectral sensitivity of LC-ToF-MS, direct injection of aqueous samples offers the potential to identify other non-targeted reaction products that may have otherwise been lost in isolation or purification steps. For example, in this work, trace levels of DOPA were identified in some experiments with confirmation using accurate-mass measurement. Finally, in more complex matrices characteristic of biological fluids or organic matter-rich natural waters, the increased specificity of analysis by ToF-MS allows for greater discrimination from matrix that can potentially complicate the use of some UV or fluorescence-based detection methods.

### Pyrite-mediated hydroxyl radical formation

The mechanisms of **^.^**OH production in pyrite slurries are not certain, yet appear important for understanding the reaction it undergoes with Phe and other organic compounds. For example, if **^.^**OH_(*ads*) _is the primary source of **^.^**OH involved in Phe reactions, and Phe reacts at the pyrite surface, then the dependence of ***R*_o _**on [Phe]_o _might be appropriately described by a L-H surface catalysis model. However, if the primary source of **^.^**OH is derived in solution by Fenton-like reactions, then there needs to be a reaction mechanism to describe the nonlinear response of ***R*_o _**with varying [Phe]_o _(Figure 4).

Any surface catalyzed reactions would most likely require the production of adsorbed **^.^**OH which may be formed through cathodic reduction of H_2_O_2 _at the pyrite surface (Equation 1) [6], or through Fe(II)/Fe(IV) electron-transfer reactions with water at surface defect-sites [8]. However, it has been argued recently that surface-defect sites in oxygenated slurries are less likely to form **^.^**OH than the Fenton reaction in solution [6]. Reaction of Phe directly at the pyrite surface with **^.^**OH_(*ads*) _seems unlikely to be a major contributor to its overall loss as Phe, Tyr, and soluble probes used in prior studies of pyrite mediated reactions [14] do not measurably adsorb to the surface (Figure 2). However, desorption of **^.^**OH_(*ads*) _may occur and resulting reactions (i.e. with Phe) could be limited to diffuse boundary layers when sufficient substrates are within a limited distance from the surface [30]. Turchi and Ollis [30] showed that such boundary layer reactions could explain kinetic behavior consistent with the L-H model, providing one possible mechanism for near surface reactions that cannot be completely ruled out.

The possibility that higher Phe levels could affect the catalytic properties of pyrite seems unlikely as in the absence of observed adsorption of Phe, its surface coverage would be very low, and decrease with increasing pyrite loading. Furthermore, there was no observed effect to reaction rates with the addition of excess Fe^2+ ^(see below), consistent with Phe not affecting the availability of iron in solution needed for the Fenton reaction.

Support for **^.^**OH formation via the Fenton reaction in the aqueous phase (Equation 2) is much stronger for a number of reasons. For example, dissolved H_2_O_2 _can be observed accumulating in pyrite slurries at concentrations relevant to this and other studies (micromolar-range) when the Fe^2+ ^in solution is chelated by EDTA, inhibiting the Fenton reaction [6,16,29]. Ferrous iron in solution also acts as a catalyst in the Fenton reaction and has been measured elsewhere to be in excess of H_2_O_2 _[29]. To confirm that ferrous iron is present in excess, experiments with 50 g/L pyrite slurries containing excess ammonium ferrous sulfate (500 μM) were incubated with 100 μM Phe. Results indicated no difference in initial or overall rates of Phe degradation when compared to a pyrite solution without iron addition (see Additional File 1, Figure S2). Therefore, there is sufficient Fe^2+ ^in pyrite slurries to suggest the **^.^**OH production is limited to the rate of production and release of H_2_O_2_, and thus by extension, the production rate of H_2_O_2 _at the pyrite surface affects the kinetics of Phe loss (and its degradation products).

### Kinetics of Phenylalanine loss

The reaction of **^.^**OH and Phe is described by the following second-order rate equation:

(4) $$-\frac{\text{d}\left[\text{Phe}\right]}{\text{dt}}=k_{\text{Phe}}\;\left[\text{Phe}\right]\;\left[\cdot \text{OH}\right]$$

Where ***k*_Phe _**is the second-order rate constant and is equivalent to 6.5 × 10^9 ^M^-1 ^s^-1 ^(as mentioned in the Results section). However, it is shown (e.g. in Figures 2, 3, 4) that -d[Phe]/d**t **does not vary proportionally with [Phe]_o_, and plateaus at higher concentrations. We suggest that the most likely mechanism for these observations is that as [Phe]_o _increases, there is a near-proportional decrease in [**^.^**OH] in the aqueous phase. This situation contrasts with more typical experiments monitoring the production of **^.^**OH or its reaction products, where either excess reactant is used and product determined, or in cases where the reactant is added at low levels in comparison to other species in solution that are sinks for **^.^**OH. This balance is constrained by the limited production rate (or flux) of **^.^**OH at any given time. To support this hypothesis we present a conceptual model that can explain most of the results from this study using three primary assumptions.

Assumption 1 asserts that the ***^.^**OH flux is constant over the time course of the experiment*. This is supported by evidence that H_2_O_2 _production has been shown to be proportional to pyrite loading [29], and does not react with substrates such as adenine [14] and Phe. If the rate of **^.^**OH formation is assumed to be constant and proportional to pyrite loading, it can be assigned a reaction coefficient that represents the pyrite reactivity (Equation 5), incorporating all factors related to formation of **^.^**OH for a particular sample of pyrite.

(5) $$R_{\cdot OH}{\;}_{\text{formation}}\;={\text{K}}_{\text{pyr}}\left[\text{pyr}\right]$$

Where **K_pyr _**has units of mol g^-1 ^hr^-1 ^(and also corresponds to the maximum rate at which Phe can be degraded). **K_pyr _**is determined for each pyrite sample to account for aforementioned variability in reaction rates (i.e. rate of **^.^**OH-formation) that was observed between different experiment sets and hypothesized to be related to conditioning of the pyrite prior to incubation.

Assumption 2 states that ***^.^**OH is in approximate steady-state *with a loss dominated by reaction with Phe (and its degradation products) and the rate of formation related to the loading and surface area of pyrite. The rate of decay for each reactant product through time is related to its individual rate constant and concentration, and with **^.^**OH formation assumed constant in Equation 5, steady-state requires the loss of **^.^**OH be constant. Thus the combined loss of total reactants must also remain constant.

(6) $$\begin{array}{l} \;\frac{\text{d}[\cdot \text{OH}]}{\text{dt}}=K_{pyr}[\text{pyr}]-\\ {}[\cdot \text{OH}](k_{\text{Phe}}[\text{Phe}]+k_{\text{Tyr}}[\text{Tyr}]+\\ \;\;\;\;\;\;\;\Sigma (k_{\text{i}}[\text{i}]))=0 \end{array}$$

Where ***k*_Tyr _**and ***k*_i _**are the second-order rate constants for the reaction of **^.^**OH with Tyr and other degradation intermediates, collectively Σ *i*, respectively.

Equation 6 represents a balance of production and loss of **^.^**OH, but also underscores that many degradation products of Phe that are likely important for describing the kinetics are unaccounted for. The degradation intermediates compete with Phe, Tyr, and each other for reaction with the limited **^.^**OH until completely oxidized to carbon dioxide (CO_2_). Therefore, the third simplifying assumption states that *the sum of all oxidizable reactants is equal to [Phe]_o _*(i.e. [Phe]_t _+ [Tyr]_t _+ Σ[*i*]_t _≈ [Phe]_o_) for most of the time course of the experiments (i.e. where Phe was still readily detectable (≥10% Phe remaining)). In addition, it is also assumed that the second-order rate constants of those products with **^.^**OH are all relatively high and similar to each other (i.e. ***k*_Phe _**≈ ***k*_Tyr _**≈ ***k*_i_**). Similar approaches have been used to simplify complex competitive rate equations in other studies involving surface-catalyzed **^.^**OH-mediated reactions [31]. Therefore, a simplified approximation of Equation 6 is represented in Equation 7.

(7) $$\begin{array}{c} \frac{\text{d}\left[\cdot \text{OH}\right]}{\text{dt}}=K_{pyr}\left[\text{pyr}\right]-\\ \;\;k_{\text{Phe}}{\left[\text{Phe}\right]}_{\text{o}}\left[\cdot \text{OH}\right]=0 \end{array}$$

Which can also be rewritten in terms of [**^.^**OH]

(8) $$\left[\cdot \text{OH}\right]=\frac{{\text{K}}_{\text{pyr}}\left[\text{pyr}\right]}{k_{\text{Phe}}{\left[\text{Phe}\right]}_{\text{o}}}$$

With substitution of Equation 8 into Equation 4, Phe loss can now be described with a rate expression that is a function of both pyrite loading and [Phe]_o_.

(9) $$-\frac{\text{d}\left[\text{Phe}\right]}{\text{dt}}=\frac{{\text{K}}_{\text{pyr}}\left[\text{pyr}\right]}{{\left[\text{Phe}\right]}_{\text{o}}}\left[\text{Phe}\right]$$

Note that in Equation 9, **K_pyr _**[pyr]/[Phe]_o _is analogous to the experimentally determined pseudo first-order rate constant ***k' ***defined in Equation 3, where ***k' ***was found to be proportional to pyrite loading and inversely related to [Phe]_o _(at [Phe] ≥ 30μM).

(10) $$k\prime =\frac{{\text{K}}_{\text{pyr}}\left[\text{pyr}\right]}{{\left[\text{Phe}\right]}_{\text{o}}}$$

Therefore, **K_pyr _**can be derived experimentally for a given pyrite sample and allows for the modeling of Phe and Tyr concentrations through time.

### Modeling Phenylalanine and Tyrosine concentrations through time

#### Determination of K_pyr_

To account for the differences in reactivity among pyrite samples, a unique value for **K_pyr _**was determined for experiments A, B, D, and E using all kinetic data collected. As described in the Results section, the temporal loss of Phe generally fits an exponential function well where ***k' ***is the slope of ln[Phe] versus **t**. As per Equation 10, **K_pyr _**is then derived from the slope of ***k' ***versus [pyr]/[Phe]_o_. The measured data were described by linear correlations between ***k' ***and [pyr]/[Phe]_o _(see Additional File 1, Figure S3), and an averaged **K_pyr _**was estimated for each experiment (A, B, D, and E) by setting the y-intercepts to zero.

#### Phenylalanine loss

Integration of Equation 9 allows for the modeling of Phe loss through time.

(11) $${\left[\text{Phe}\right]}_{t}={\left[\text{Phe}\right]}_{\text{o}}\left(e^{-\frac{{\text{K}}_{\text{pyr}}\left[\text{pyr}\right]}{{\left[\text{Phe}\right]}_{\text{o}}}t}\right)$$

**Figure 10:**
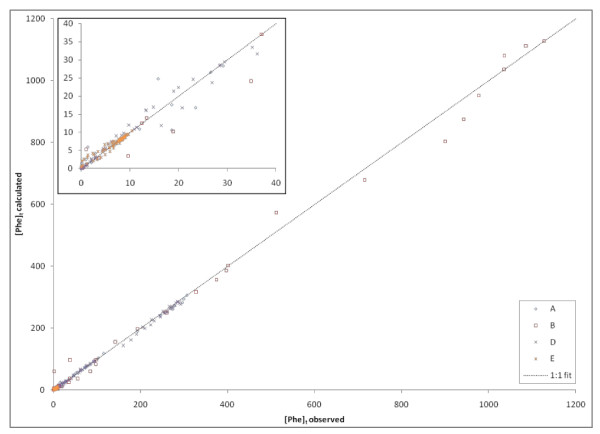
**Modeled versus observed Phe concentrations for 4 different sets of experiments**. Calculated concentrations of Phe were derived from Equation 10 using the **K_pyr _**calculated for experiment sets A, B, D, and E. The model values fit the observed data well despite the observed variability in **K_pyr _**among batches of pyrite.

Observed Phe data from Experiments A, B, D and E along with predicted values (modeled with Equation 11) are plotted in Figure 10. Comparisons of predicted versus observed [Phe]_t _was fit by a linear regression model with a slope of 0.985 with an R^2 ^of 0.996.

#### Tyrosine production and loss

The kinetics of Tyr formation and loss are governed by consecutive reactions, and using the assumptions described above, the rate of change can be represented in Equation 12.

(12) $$\begin{array}{l} \frac{\text{d}[\text{Tyr}]}{\text{dt}}={\text{K}}_{\text{pyr}}[\text{pyr}]\cdot \\ \;\;\;\left(\frac{\alpha \;k_{\text{Phe}}[\text{Phe}]-k_{\text{Tyr}}[\text{Tyr}]}{k_{\text{Phe}}[\text{Phe}]+k_{\text{Tyr}}[\text{Tyr}]+\sum (k_{\text{i}}[\text{i}]))}\right) \end{array}$$

Where **α **is the fraction of the Phe reaction products corresponding to Tyr. For modeling purposes, **α **was estimated to be 0.5, which was within the range of yields determined from initial rate data. Again, assumptions made in this model consolidate the competitive factor that affect Phe and other products that react with **^.^**OH in the slurry. Thus Equation 12 can be simplified to:

(13) $$\frac{\text{d}\left[\text{Tyr}\right]}{\text{dt}}=\frac{{\text{K}}_{\text{pyr}}\left[\text{pyr}\right]}{{\left[\text{Phe}\right]}_{\text{o}}}\cdot \left(\alpha \left[\text{Phe}\right]-\left[\text{Tyr}\right]\right)$$

Integration of Equation 13 yields:

(14) $${\left[\text{Tyr}\right]}_{\text{t}}=\alpha \;{\text{K}}_{\text{pyr}}\left[\text{pyr}\right]\text{t}\;\left(e^{-\frac{k_{\text{pyr}}\left[\text{pyr}\right]}{{\left[\text{Phe}\right]}_{\text{o}}}}\right)$$

**Figure 11:**
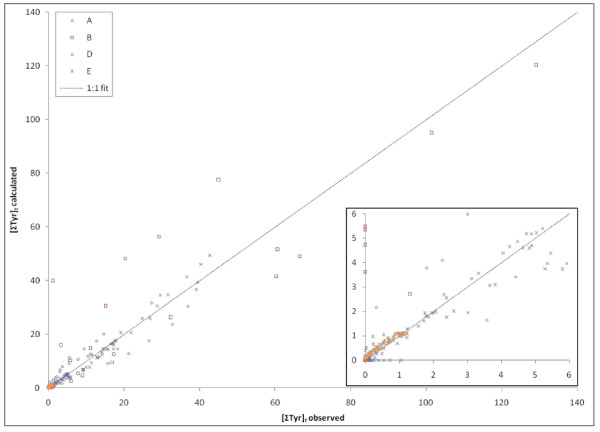
**Modeled versus observed ΣTyr concentrations for 4 different sets of experiments**. Corresponding to the data for Phe shown in Figure 10, concentrations of ΣTyr were derived using Equation 14, the **K_pyr _**calculated for each experimental set, and a value of 0.5 for α. Note that for points where values are predicted but not measured for Tyr, there was an apparent lag in Phe degradation at the first time point (see inset). Similarly, several instances when no Tyr is predicted to be present, low-levels were observed corresponding to later time points in the incubation where the vast majority of Tyr had been degraded.

Equation 14, based on data for Phe loss, provided predictions of [Tyr]_t _that agreed with measured values from Experiments A, B, D, and E reasonably well (Figure 11). A linear regression fit of points in Figure 11 has a slope of 1.18 with an R^2 ^of 0.888.

This simplified model is able to predict much of the change in Phe and Tyr through time when the specific **K_pyr _**for a pyrite sample is determined. Importantly, it allows for most of the observed dependence of [Phe]_o _and pyrite loading to be accounted for while describing the apparent first-order dependence of Phe loss through time. Based on this analysis, there is no need to consider potential surface reactions between **^.^**OH and Phe to model the hyperbolic function of ***R*_o _**at higher [Phe]_o _(Figure 4), normally ascribed to surface catalytic reactions. However, it should be pointed out that this simple model does not describe the decrease in ***R*_o _**observed at lower [Phe]_o _as well. Although this may be related to the paucity of initial measurements when Phe degrades rapidly at lower [Phe]_o_, it does seem likely that initial rates are lower when [Phe]_o _is low (<30 μM). We hypothesize that at low [Phe]_o_, there are other reactions that compete for **^.^**OH with Phe and other products. Whether or not such reactions occur at the pyrite surface or with trace inorganic reactants in solution is uncertain and beyond the scope of this work.

A more detailed numerical simulation was also developed to compare to results from the simplified model presented above (Equations 11 and 14), and to provide greater flexibility for testing the effect of assumptions concerning reaction pathways and relative rates of multiple products with **^.^**OH (see Additional File 1, *Numerical simulation *section). Values of second-order rate constants for different reaction products (i.e. Tyr and DOPA) and the fraction of Phe-to-ΣTyr conversions (reaction yield, or **α**) were adjusted to test basic assumptions made above. The assumption of constant **^.^**OH-flux was not changed. Figures in the Additional File 1 show that calculations made with ***k*_Phe _**= ***k*_Tyr _**= ***k*_DOPA _**and **α **= 0.5 (50% conversion of Phe-to-ΣTyr) offer the closest match to observed and modeled data for most incubations.

## Conclusion

Loss of Phe varied in pyrite slurries at rates that were first-order in pyrite loading and pseudo first-order dependent on Phe as a function of time; whereas the rate of Phe loss was much less-than first-order in [Phe]_o_. The data for the loss of Phe as well as the production and loss of Tyr products could be described well by a mechanistically-based kinetic model that reconciles the observations concerning the initial concentration dependence of Phe. The competitive effects of degradation products on reactions of molecular probe have been included in kinetic descriptions; as such products become important when substrate is not added in great excess. Not limited to experiments with pyrite, there have been few studies that have considered that observed exponential decay of reactants may be due to increased competition for available **^.^**OH [31], rather than due to true first-order behavior. In systems where the flux of **^.^**OH is likely the rate limiting step, observed kinetics will depend on whether the relative amounts of competing reactants change as a function of time. The [ΣTyr]/[Phe]_o _and [Tyr-isomer]/[ΣTyr] ratios were consistent throughout experiments, with total-Tyr formation estimated to be about 50% of the Phe conversion via **^.^**OH. The use of Phe and its **^.^**OH-specific products is argued to be a useful probe that should be further developed for the study of the mechanisms of pyrite and other mineral-mediated reactions, and has the potential to be a valuable tool for the study of **^.^**OH reactions in a range of other systems with more complex matrices.

## Competing interests

The authors declare that they have no competing interests.

## Authors' contributions

SCF conducted the experiments, interpreted data, and drafted the manuscript. MAAS participated in experimental design, provided financial support and facilities, and assisted with manuscript preparation and revision. BJB participated in the design of experiments, data interpretation, and helped draft the manuscript. All authors read and approved the final manuscript.

## Supplementary Material

Additional File 1 **Supporting information for reactions of phenylalanine in pyrite slurries**. This file includes figures illustrating the constancy of Tyr-isomer ratios as afunction of time; the effects of addition of ferrous iron on Phe reactions in pyrite slurries; and the data and model used to calculate **K_pyr _**in a given experiment. Finally a description of a numerical simulation model is described with examples of the effects of model variables when both analytical and simulation models are compared to observed data. Click here for file
