# Supplementary material for: Phenylalanine as a hydroxyl radical-specific probe in pyrite slurries
Source: Geochem Trans. 2012 Feb 7;13:3. doi: 10.1186/1467-4866-13-3 (PMC3348026; doi:10.1186/1467-4866-13-3)
Supplement: Additional File 1 — Supporting information for reactions of phenylalanine in pyrite slurries. This file includes figures illustrating the constancy of Tyr-isomer ratios as afunction of time; the effects of addition of ferrous iron on Phe reactions in pyrite slurries; and the data and model used to calculate Kpyr in a given experiment. Finally a description of a numerical simulation model is described with examples of the effects of model variables when both analytical and simulation models are compared to observed data. [file 1467-4866-13-3-S1.PDF]

Supplemental Information:

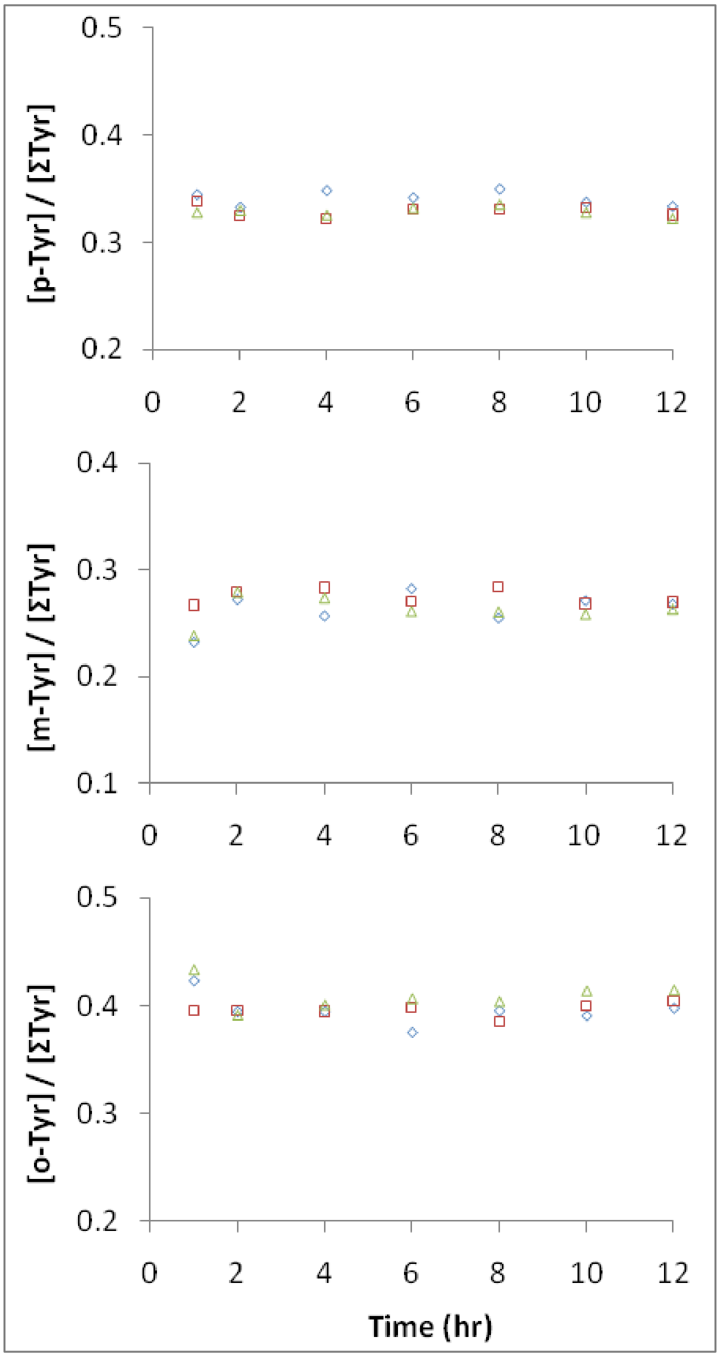

Figure S1 **Tyr-isomer ratios relative to ΣTyr during 100 µM Phe degradation (Exp. D).** The proportions of each Tyr isomer are uniform among samples and are independent of $[Phe]_o$ and pyrite loading, with *p*-Tyr as 32%, *m*-Tyr as 28%, and *o*-Tyr as 40% of the ΣTyr. These results from experiment D of three different sets of 100 µM Phe experiments with **25 g/L** (◇), **50 g/L** (□), **100 g/L** (∆) pyrite loading are characteristic of observed experimental results where Phe was still present.

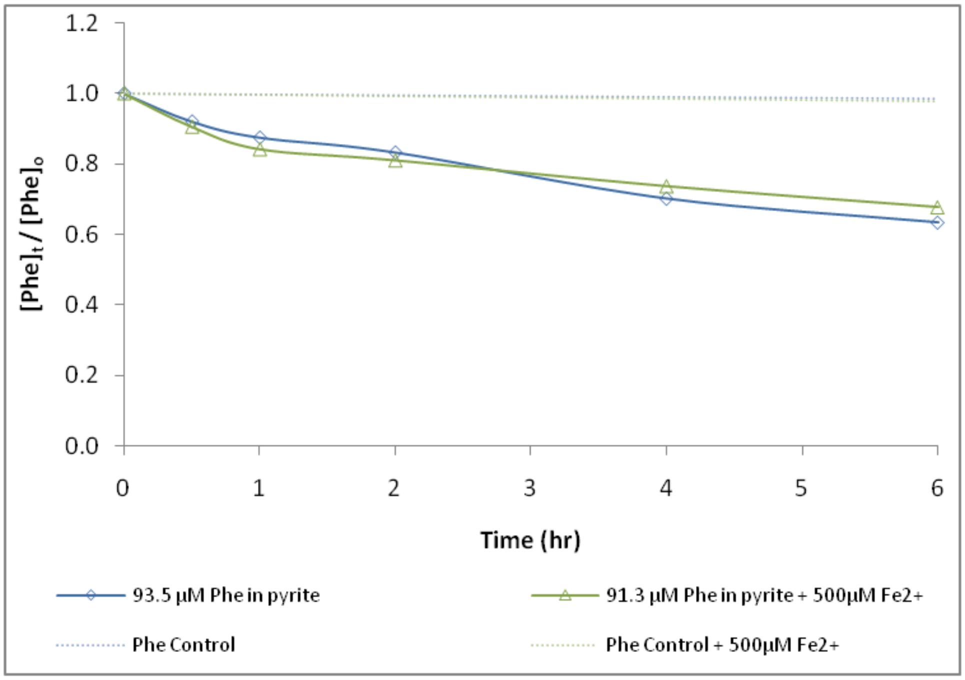

Figure S2 **Loss of Phe in 50 g/L pyrite with and without additional 500 µM Fe^{2+}.** Results from incubations of Phe in 50 g/L pyrite with and without 500 µM Fe^{2+} (in the form or Mohr's salt) indicate that there is no significant differences in the rate of Phe loss over time. No loss of Phe was observed in two different control incubations without pyrite, and the production of expected levels of Tyr were also determined (data not shown). This confirms that ferrous iron is not the limiting factor in the formation of ·OH (via the Fenton reaction) in pyrite slurries.

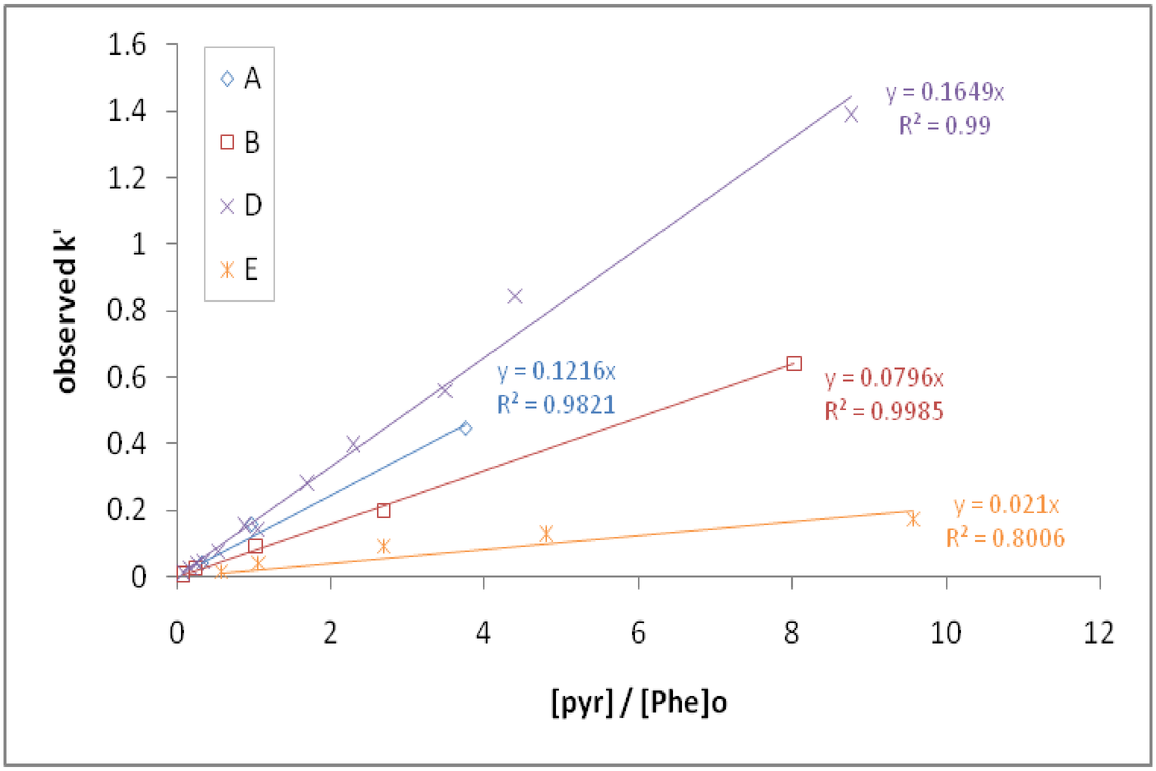

Figure S3 **Determination of estimated pyrite reactivity constant, K_{pyr} , for a given pyrite sample.** Pseudo first-order rate constant ***k'*** (obtained empirically by fitting ln[Phe] versus **t**) versus pyrite loading over $[Phe]_o$; the resulting slopes correspond to the $\mathbf{K_{pyr}}$ of the sample of pyrite in a given experiment (A, B, D, or E) that can be used to model the data.

Numerical simulation

In contrast to the simplified model discussed in the main text, numerical simulations formulated to test some of the assumptions do not necessarily assume that $[Phe]_t + [Tyr]_t + \Sigma[i]_t \approx [Phe]_o$ (i.e. formation of CO_2 is permitted and removed from the equation). CO_2 formation decreases the reactant pool of reactive intermediates competing for $^{\cdot}OH$. Two hypothesized reaction pathways (Figures S4 and S5) were compared to test the differences between a reaction pathway where Phe is completely degraded in just four steps to CO_2 (short pathway, Figure S4), and one requiring a greater number of reactions and intermediates (long pathway, Figure S5). Reaction Figure S5 would seem more reasonable given the number of carbons in Phe, Tyr, and DOPA that would need to be fully oxidized to CO_2.

$$\begin{array}{ccccccc} Phe & \stackrel{\alpha}{\rightarrow} & Tyr & \stackrel{\beta}{\rightarrow} & DOPA & \stackrel{\gamma}{\rightarrow} & i_1 \rightarrow CO_2 \\ \downarrow & & \downarrow & & \downarrow & & \\ i_2 & & i_5 & & i_7 & & \\ \downarrow & & \downarrow & & \downarrow & & \\ i_3 & & i_6 & & CO_2 & & \\ \downarrow & & \downarrow & & & & \\ i_4 & & CO_2 & & & & \\ \downarrow & & & & & & \\ CO_2 & & & & & & \end{array}$$

Figure S4 Hypothetical reaction scheme with nine reaction intermediates "short pathway". Theoretical "short" pathway for the reaction of ˙OH with Phe and three subsequent intermediates where all reactants are susceptible to ˙OH reaction oxidation until CO_2 is formed and removed from competition.

$$\begin{array}{ccccccc} Phe & \stackrel{\alpha}{\rightarrow} & Tyr & \stackrel{\beta}{\rightarrow} & DOPA & \stackrel{\gamma}{\rightarrow} & i_1 \rightarrow i_2 \rightarrow i_3 \rightarrow i_4 \rightarrow i_5 \rightarrow CO_2 \\ \downarrow & & \downarrow & & \downarrow & & \\ i_6 & & i_{13} & & i_{19} & & \\ \downarrow & & \downarrow & & \downarrow & & \\ i_7 & & i_{14} & & i_{20} & & \\ \downarrow & & \downarrow & & \downarrow & & \\ i_8 & & i_{15} & & i_{21} & & \\ \downarrow & & \downarrow & & \downarrow & & \\ i_9 & & i_{16} & & i_{22} & & \\ \downarrow & & \downarrow & & \downarrow & & \\ i_{10} & & i_{17} & & i_{23} & & \\ \downarrow & & \downarrow & & \downarrow & & \\ i_{11} & & i_{18} & & CO_2 & & \\ \downarrow & & \downarrow & & & & \\ i_{12} & & CO_2 & & & & \\ \downarrow & & & & & & \\ CO_2 & & & & & & \end{array}$$

Figure S5 Hypothetical reaction scheme with 25 reaction intermediates "long pathway". Theoretical "long" pathway for the reaction of ˙OH with Phe and seven subsequent intermediates where all reactants are susceptible to ˙OH reaction oxidation until CO_2 is formed and removed from competition. This pathway seems more realistic as there are many carbon atoms to oxidize before complete mineralization of Phe can be achieved.

To assess the effect of varying the individual rate constants for each reactant, the simulation solves competitive equations (analogous to Equation 12 for Tyr) iteratively for Phe, Tyr, DOPA, and i_n (where n can equal 1-7 or 5-23 depending on the pathway). The percentage of total $^{\cdot}$OH-flux that is consumed by an individual reactant is determined by the product of its relative rate constant, ***f*** (defined here as the ratio of the second-order rate constant of a reactant relative to the rate constant for Phe (e.g. $\boldsymbol{f_i} = \boldsymbol{k_i} / \boldsymbol{k_{Phe}}$)), and its concentration divided by the sum of analogous terms for all competing reactants.

Calculations for the numerical simulation were done in Microsoft® Excel 2007 using Equations S1 for [Phe] and S2 for [ΣTyr] at each time interval. Concentrations of additional degradation products (represented as "i_n" (includes DOPA in the Equations)) were calculated with similar formulas to for each species represented by Figures S4 and S5 ("short" and "long" degradation pathways, respectively).

$$[\mathrm{Phe}]_t = [\mathrm{Phe}]_{t-1} - \left([\cdot\mathrm{OH}]_t \left(\frac{k_{\mathrm{Phe}}[\mathrm{Phe}]_{t-1}}{k_{\mathrm{Phe}}[\mathrm{Phe}]_{t-1} + k_{\mathrm{Tyr}}[\mathrm{Tyr}]_{t-1} + \Sigma\left(k_{i_n}((1 - \%\text{-to-}\mathrm{CO_2})[\mathrm{i_n}]_{t-1})\right) + \mathbf{K}}\right)\right) \quad \text{(S1)}$$

$$[\mathrm{Tyr}]_t = \alpha\left([\mathrm{Phe}]_t - [\mathrm{Phe}]_{t-1}\right) + [\mathrm{Tyr}]_{t-1} - \left([\cdot\mathrm{OH}]_t \left(\frac{k_{\mathrm{Tyr}}[\mathrm{Tyr}]_{t-1}}{k_{\mathrm{Phe}}[\mathrm{Phe}]_{t-1} + k_{\mathrm{Tyr}}[\mathrm{Tyr}]_{t-1} + \Sigma\left(k_{i_n}((1 - \%\text{-to-}\mathrm{CO_2})[\mathrm{i_n}]_{t-1})\right) + \mathbf{K}}\right)\right) \quad \text{(S2)}$$

Analytical results and estimated $\mathbf{K_{pyr}}$ from Experiment D were compared to predictions by both analytical solution of the model presented above (Equations 11 and 14; Figure S6) and numerical simulations described here (Figure S7). Predictions from the simplified model was examined first alongside observed data points for Phe and Tyr for 29.5 μM $[\mathrm{Phe}]_o$ in 50 g/L pyrite loading (Figure S6A) and 94.5 μM $[\mathrm{Phe}]_o$ in 100 g/L pyrite loading (Figure S6B), respectively.

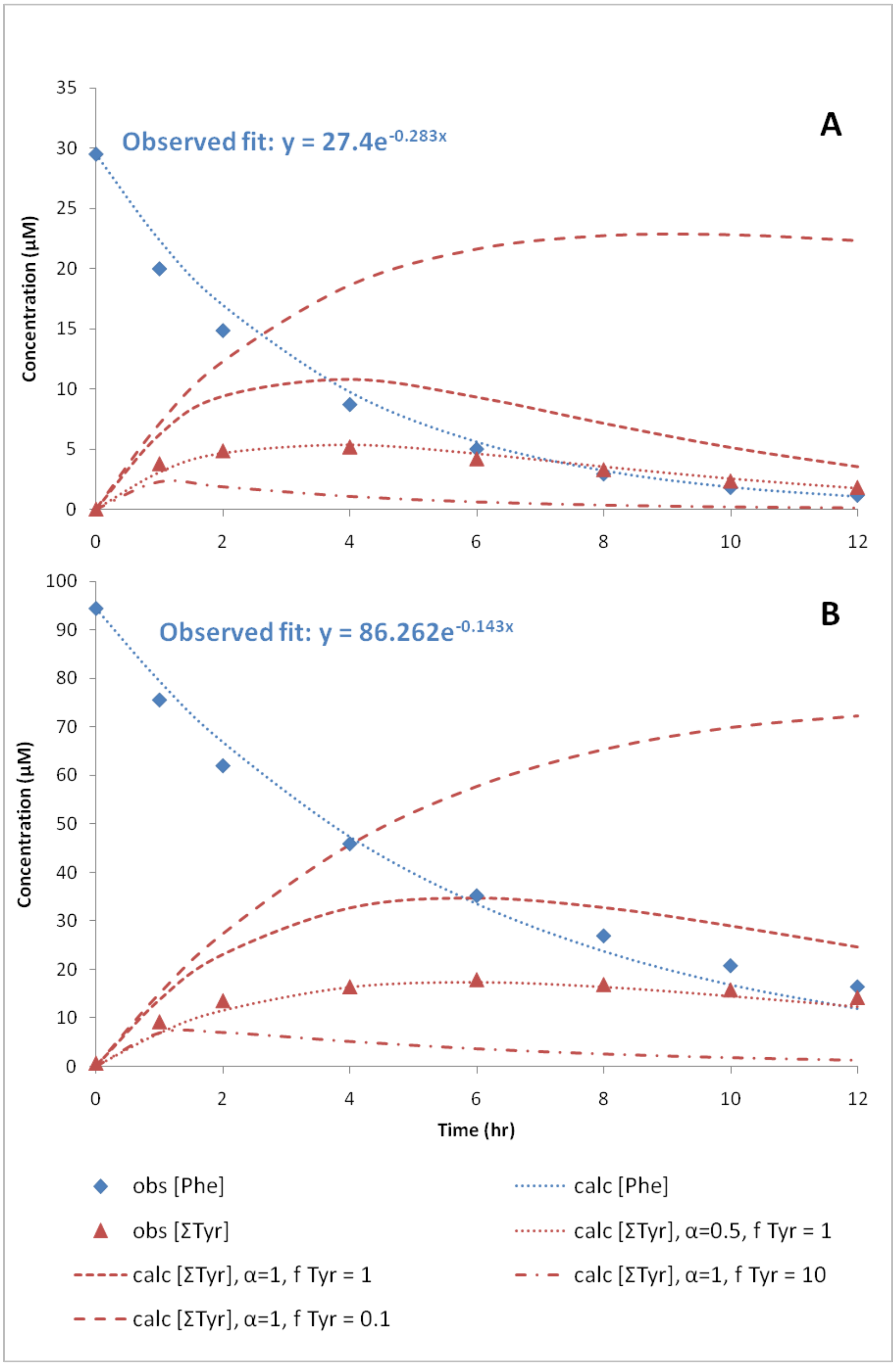

Figure S6 Calculations for the simplified model based on experimental data. Kinetic data from experiment D is plotted with $[Phe]_o$ of 29.5 µM (A) and 94.5 µM (B) and pyrite loading of 50 g/L and 100 g/L, respectively. Values of $\boldsymbol{\alpha}$ are either 0.5 or 1 and values of $\boldsymbol{f}_{\mathbf{Tyr}}$ were set to 0.1, 1, or 10; values of $\boldsymbol{f}_{\mathbf{i}}$ were all assumed to equal 1 for the purposes of this exercise.

The models showed no change in the kinetics of Phe loss when $\boldsymbol{\alpha}$ or $\boldsymbol{f_{Tyr}}$ were varied because the underlying assumptions that led to Equation 11 have not been changed. However, ΣTyr calculated when $\boldsymbol{\alpha}$ = 0.5 or 1 and $\boldsymbol{f_{Tyr}}$ = 0.1, 1, or 10 were dramatically different, and reveal the closest fit to the actual data when $\boldsymbol{\alpha}$ = 0.5 and $\boldsymbol{f_{Tyr}}$ = 1. Predicted ΣTyr, when $\boldsymbol{k_{Tyr}}$ did not equal $\boldsymbol{k_{Phe}}$, were not possible with Equation 14. Instead, integration of a more general rate expression for consecutive reactions was used (Equation S3).

$$[\mathrm{Tyr}]_{\mathrm{t}} = \alpha\,[\mathrm{Phe}]_{\mathrm{o}} \left(\frac{\frac{\mathrm{K_{pyr}}\,[\mathrm{pyr}]}{[\mathrm{Phe}]_{\mathrm{o}}}}{k'_{\mathrm{Tyr}} - \frac{\mathrm{K_{pyr}}\,[\mathrm{pyr}]}{[\mathrm{Phe}]_{\mathrm{o}}}} \right) \left(e^{-k'_{\mathrm{Tyr}}\,\mathrm{t}} - e^{-\frac{\mathrm{K_{pyr}}\,[\mathrm{pyr}]}{[\mathrm{Phe}]_{\mathrm{o}}}\,\mathrm{t}} \right) \tag{S3}$$

The numerical simulation was used to test the assumption $\boldsymbol{k_{Tyr}} \approx \boldsymbol{k_{Phe}}$ made in the simplified first-order model for [Tyr] through time (Equations 13 and 14). Assuming that $\boldsymbol{k_{Tyr}} \neq \boldsymbol{k_{Phe}}$ results in a different integration for determining [Tyr] (Equation S3). However, when simulations were run with a realistic range of $\boldsymbol{k_{Phe}}$ / $\boldsymbol{k_{Tyr}}$, the predicted [Tyr] did not appreciably differ from predictions using the assumption that the rate constants were equal (Equation 14).

Simulations were then examined in Figure S7 with the same $[\mathrm{Phe}]_o$ and pyrite loadings shown in Figure S6. Figure S7 also considers the short and long pathways (Figures S4 and S5) and shows simulations of CO_2 concentration formed over time. In agreement with the predictions from the model (Figure S6), an excellent fit to the observed data is also represented by the simulations that set $\boldsymbol{\alpha}$ = 0.5 and $\boldsymbol{f_{Tyr}}$ = 1 (Figures S7A & E) with the powers of the exponential fits similar. Phe concentration through time when $\boldsymbol{\alpha}$ is increased to 1 (Figures S7B & D) are identical to those determined when $\boldsymbol{\alpha}$ = 0.5 (Figures S7A & E); however, Tyr concentration at each time point doubles as a result of $\boldsymbol{\alpha}$ = 1 channeling all Phe degradation through Tyr.

Figures S7C & G illustrate the effect of increasing the reactivity of Tyr in comparison to Phe and represent a $\boldsymbol{k_{Tyr}}$ that is 10-times faster than $\boldsymbol{k_{Phe}}$ ($\boldsymbol{f_{Tyr}}$ = 10) when all the Phe in the system forms Tyr ($\boldsymbol{\alpha}$ = 1). Not only is measured $[\mathrm{Tyr}]_t$ under-predicted by the model, but $[\mathrm{Phe}]_t$ is over-predicted as expected, the latter due to increased competition for $^{\cdot}OH$ even at lower concentrations of Tyr. Similarly, where $\boldsymbol{k_{Tyr}}$ is one-tenth the value of $\boldsymbol{k_{Phe}}$ ($\boldsymbol{f_{Tyr}}$ = 0.1) and $\boldsymbol{\alpha}$ = 1, the simulated results show a faster, near zero-order loss of Phe over much of the time and a relatively large build-up of Tyr (Figures S7D & H). Compared to other the simulations where

CO_2 production is only observed when modeling the short pathway, CO_2 evolves more rapidly when $\boldsymbol{f}_{\mathbf{Tyr}}$ is small, and appreciable amounts are even projected to form over the long pathway.

Overall, there are not significant differences in the loss of Phe through time when the short and long pathways are compared with the assumptions used. Variations in $\boldsymbol{\alpha}$ and $\boldsymbol{f}_{\mathbf{Tyr}}$ can change the apparent kinetics in both the numerical simulations and the analytical models. Accumulation of CO_2 in the short pathway is much more rapid; however, it does not appreciably influence the loss of Phe because the pool of oxidizable reactants is not greatly depleted over the timescale of Phe degradation. This is also generally the case for Tyr, with the notable exception of the simulation illustrated in Figure S7D corresponding to greater amounts of CO_2 produced and loss of competing reactants.

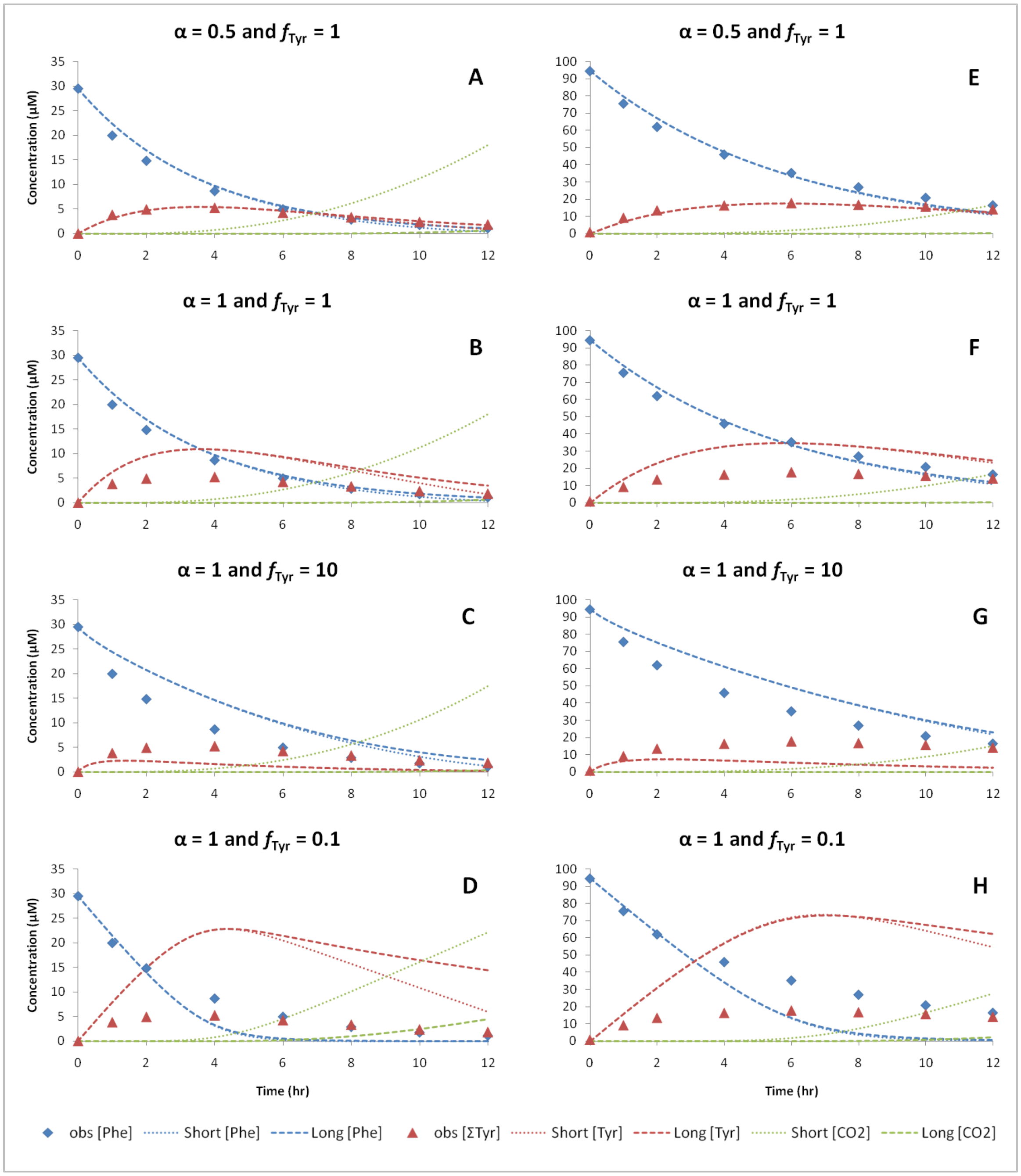

Figure S7 Simulations of kinetic curves for experiment D is presents with $[Phe]_o$ of 29.5 µM (A-D) and 94.5 µM (E-H) and pyrite loading of 50 g/L and 100 g/L, respectively. Values of **α** are either 0.5 or 1 and values of $\boldsymbol{f_{Tyr}}$ was set to 0.1, 1, or 10.
